# Comparison of scoring systems for bleeding in open cardiac surgery patients

**DOI:** 10.55730/1300-0144.6039

**Published:** 2025-04-17

**Authors:** İpek YAKIN, Çağrı DÜZYOL, İlke DOLĞUN, Ahmet YÜKSEK, Kemal Tolga SARAÇOĞLU

**Affiliations:** 1 Department of Anesthesiology and Reanimation, Kocaeli City Hospital, Kocaeli, Turkiye; 2 Department of Cardiovascular Surgery, Kocaeli City Hospital, Kocaeli, Turkiye; 3 Department of Anesthesiology and Reanimation, Faculty of Medicine, İstinye University Medicalpark Gaziosmanpaşa Hospital, İstanbul, Turkiye; 4 Department of Anesthesiology and Reanimation, Faculty of Medicine, Qatar University, Doha, Qatar

**Keywords:** Bleeding, blood transfusion, perioperative care, open-heart surgery, anesthesia

## Abstract

**Background/aim:**

The aim of our study was to determine which preoperative bleeding risk scoring system is more sensitive in predicting perioperative transfusion requirement in patients undergoing open-heart surgery.

**Materials and methods:**

This is a retrospective single-center cohort study. Seven scoring systems (TRACK, PAPWORTH, WILL-BLEED, CRUSADE, ACTION, TRUST, ACTA-PORT) were used to predict the likelihood of perioperative erythrocyte suspension (ES) transfusion requirement.

**Results:**

Four hundred patients were enrolled in the study. Age, creatinine level, and diagnoses of diabetes mellitus and hypertension were significantly higher in patients who required ES (p < 0.05). In addition, ejection fraction percentages and hemoglobin and hematocrit levels were significantly lower (p < 0.05). Except for PAPWORTH; ACTION, ACTA-PORT, WILL-BLEED, TRACK, TRUST, and CRUSADE scores were higher in the ES group (p < 0.05), but the most predictive scoring system for ES use was TRUST.

**Conclusion:**

The ACTION and ACTA-PORT systems were also found to significantly predict ES use, but the WILL-BLEED, TRUST, and TRACK systems were found to be more predictive of bleeding and ES transfusion requirement in CABG operations. Furthermore, low EF, Hb, and Hct levels, higher creatinine levels, and the presence of DM were identified as individual risk factors for perioperative bleeding, apart from the scoring systems.

## 1. Introduction

In coronary revascularization surgeries, the average mortality rate can reach as high as 6.2%. This rate can reach 10% when valve surgeries are included and up to 20% following severe bleeding. [1]. Excessive bleeding following cardiac surgery remains a complex clinical problem despite advances in surgical technique, anesthetic management, and critical care [2].

Perioperative bleeding may result from several factors, including new-generation antithrombotic drugs [3,4]. This is particularly important for patients undergoing coronary artery bypass grafting (CABG) because they are often treated with potent antithrombotic drugs preoperatively [3]. Identifying patients with increased perioperative bleeding risk in the preoperative period may not only prevent perioperative blood loss but also reduce the use of blood products [3,5]. Even though it is believed that less bleeding is better and transfusion should be avoided, all patients bleed following surgery, and the point at which bleeding becomes clinically significant is not clearly defined. Therefore, a “universal classification for the definition of perioperative bleeding” was developed to clearly define the severity of bleeding [2].

Risk scoring systems have been developed to evaluate patients in terms of bleeding risk. Some of these are used for percutaneous coronary interventions [6,7], while others are used for adult cardiac surgery [3,8–10]. These scoring systems generally use similar parameters and estimate bleeding risk, but it is unclear whether some of them can be applied to coronary revascularization surgeries or which one is more predictive.

The hypothesis of our study is that all seven scoring systems tested can predict the need for erythrocyte suspension in patients undergoing coronary artery bypass grafting. Our aim is to discuss the new risk scoring and estimation systems supported by scientific studies in the literature and to identify the preoperative risk scoring system that is most sensitive in predicting the need for erythrocyte suspension.

## 2. Materials and methods

### 2.1. Study design

This study is a retrospective cohort study initiated after the approval of the Health Sciences University Hamidiye Noninvasive Studies Ethics Committee. (Ethics committee number 18/24, on 10.08.2016) (ClinicalTrial ID: NCT03846622).

During our study period, the data of 500 patients were reviewed retrospectively. Four hundred patients over the age of 18 who underwent isolated CABG with cardiopulmonary bypass between 2015 and 2018 were enrolled in our study. Patients who underwent emergent operations, valvular surgery, or off-pump surgery, and those whose data regarding the scoring systems were missing, were excluded (n = 100). Demographic and surgical data of the patients—such as age, sex, body mass index, comorbidities, the extent of surgery and bleeding, and the duration of the operation—were recorded from hospital software, patient files, and anesthesia records. These data are presented in tables.

Seven scoring systems (ACTION [7,11], ACTA-PORT [12], WILL-BLEED [12], PAPWORTH [12], TRACK [13], TRUST [14], CRUSADE [15]) were used as the most frequently compared bleeding scores in the literature for CABG and percutaneous coronary interventions to predict the likelihood of perioperative bleeding. All data from eligible patients were evaluated for each of the seven scoring systems, and the results were compared with perioperative bleeding and transfusion requirements. The scales used were summarized in the appendix.

### 2.2. Our transfusion protocol

One unit of erythrocyte suspension contains approximately 200 mL of erythrocytes. It has a total volume of approximately 300 mL including preservatives. Hematocrit is around 65%–75%. The necessity of blood transfusions was determined according to our clinical approach based on a restrictive transfusion strategy. The following method was used in transfusions: no transfusion was performed when hemoglobin (Hb) was ≥10g/dL and/or hematocrit (Hct) was ≥ 30%. Transfusion was avoided when Hb was ≥8 g/dL and/or Hct was ≥24%, unless there was a strong clinical indication. When Hb was 7–8 g/dL and/or Hct was 21%–24%, transfusion was considered based on individual assessment of tissue oxygenation. Transfusion was performed when Hb was <7 g/dL and/or Hct was <21%.

Since blood loss is reaspirated in surgeries using heart-lung pumps, and our study hypothesis focuses on transfusion requirement, total blood transfusion was chosen as the primary measurement parameter instead of perioperative bleeding.

### 2.3. Statistical analysis

For statistical analysis of the definitive data, values such as mean, standard deviation (SD), median, minimum, maximum, frequency, and rate were used. The distribution of the variables was tested using the Kolmogorov–Smirnov test. For the analysis of normally and nonnormally distributed continuous variables, the independent samples t-test and Mann–Whitney U test were used. For the analysis of categorical variables, the chi-square test was used, and the Fisher’s exact test was applied when the assumptions of the chi-square test were not met. Pearson and Spearman correlation analyses were used. Effect sizes were evaluated using ROC curve analysis and univariate and multivariate logistic regression. Due to the strength of the statistical results obtained, neither false discovery rate (FDR) correction nor Bonferroni correction was applied to reduce type I error. All statistical analyses were performed using SPSS version 22.0 (IBM Corp., Armonk, NY, USA).

## 3. Results

After excluding patients who met the exclusion criteria, 400 patients aged 33–86 years (61.81 ± 8.89 years) were enrolled in the analysis. Of these, 84 (21.0%) were female and 316 (79.0%) were male. Demographic data and the number of perioperative erythrocyte suspension (ES) transfusion units were presented in Table 1.

Among the patients, 63% had low risk according to ACTION, 86% had moderate risk according to ACTA-PORT, 55% had low risk based on WILL-BLEED, 83% had moderate risk with PAPWORTH, 67% had low risk with TRACK, 67% had moderate-to-high risk with TRUST, and 58% had very low risk with CRUSADE classifications.

In the ES+ group, patient age was statistically significantly higher (p < 0.05). There was no statistically significant difference between the ES+ and ES− groups in terms of body mass index (BMI), cross-clamp (CC) time, and cardiopulmonary bypass (CPB) time (p > 0.05). In the ES+ group, ejection fraction (EF), Hb, and Hct levels were significantly lower than in the ES− group (p < 0.05). The creatinine level was significantly higher in the ES+ group than in the ES− group (p < 0.05). The rates of diabetes mellitus (DM) and hypertension (HT) were also significantly higher in the ES+ group (p < 0.05). The rate of anemia was significantly lower in the ES+ group (p < 0.05). The rates of PAD, chronic renal failure (CRF), and anticoagulant use did not differ significantly between the groups (p > 0.05) (Table 2).

In the ES+ group, ACTION, ACTA-PORT, WILL-BLEED, TRACK, TRUST, and CRUSADE scores were statistically significantly higher (p < 0.05) (Table 3). Although the PAPWORTH score was higher in the ES+ group, the difference was not statistically significant (p = 0.112). In addition, according to our ROC analysis, the PAPWORTH score did not have sufficient power or sensitivity to predict ES use. The most predictive scoring system for ES use was TRUST (Table 4; Figure).

In the univariate model, age, EF, sex, Hb, Hct, DM, HT, anticoagulant use, and the ACTION, ACTA-PORT, WILL-BLEED, TRACK, TRUST, and CRUSADE scoring systems were statistically significant predictors of ES use (p < 0.05). In the reduced multivariable model, DM and the ACTION, TRUST, and WILL-BLEED scores were found to be statistically and independently significant predictors of ES use (p < 0.05) (Table 5).

## 4. Discussion

In this study, which evaluated the predictive power of seven scoring systems for perioperative blood transfusion requirements, the scores with the highest predictive value for ES use were WILL-BLEED, TRUST, and TRACK. Additionally, the group of patients who received erythrocyte suspension had higher age [64.96 ± 8.54 (65)], lower EF, Hb, and Hct, higher creatinine, and a higher prevalence of DM.

Cardiovascular operations are associated with relatively higher rates of transfusion of blood and blood products. Numerous hemolytic and nonhemolytic side effects of transfusion have been reported. Transfusion has been identified as a contributing factor to prolonged postoperative extubation time, sepsis, acute respiratory distress syndrome, postoperative sternal wound infection, renal failure, and mortality in cardiac surgery [16]. The first step in patient blood management is to assess individual risk and take appropriate measures to reduce it.

In our study group, ES use was not significantly higher among patients with CRF. Possible explanations for this finding include the multifactorial nature of ES utilization, the implementation of a restrictive transfusion strategy, and the fact that preoperative hemoglobin levels in CRF patients were similar to those in non-CRF patients. Additionally, limiting the study to elective surgeries may have excluded anemic or more frail CRF patients [17,18]. In contrast, a study by Eranki et al. involving 1595 patients found that dialysis, reduced creatinine clearance, and cardiogenic shock were associated with increased ES utilization [19]. Notably, the study emphasized that the combination of these risk factors further increased the likelihood of transfusion. Similarly, ES utilization did not differ significantly among patients on anticoagulant therapy. However, when examining the groups, ES transfusion was required in 1.9% versus 5.8% of patients. Although this difference was not statistically significant, the p-value approached the threshold for significance. Different results might have been obtained in larger patient populations or in groups undergoing emergency surgeries without preoperative optimization. These findings also highlight the potential contribution of preoperative optimization and elective surgical scheduling to improved perioperative outcomes.

Perioperative bleeding may lead to increased use of blood products, prolonged mechanical ventilation support, extended intensive care unit stays, and depletion of hospital sources [16]. Developing a stable algorithm for patient blood management may help clinicians adopt an optimal approach to reduce bleeding and its related complications. An effective patient blood transfusion management protocol should begin with identifying patients at high risk of bleeding in the perioperative period. It is important to take measures to avoid unnecessary blood transfusions and to reduce perioperative blood loss [18]. A scoring system for this would be particularly important in cardiac surgery patients, as they are usually treated with antithrombotic agents and therefore require effective precautions to reduce excessive perioperative bleeding. There is a need to improve various preoperative bleeding risk assessment tools. Moreover, the increasing use of machine learning has led to the need to reexamine and compare existing predictive scores. In our study, seven different scoring systems intended for this purpose were compared [20].

One of these scoring methods, named “TRUST”, was developed by Alghamdi et al. [9]. It was designed to classify heart surgery patients in terms of their need for blood transfusion, based on eight variables. This method is considered simpler and more effective compared to other complex methods. Krishna et al. [14] reported that the TRACK system could provide better perioperative blood management than TRUST. In our study, the most predictive scoring system for the need for ES transfusion was TRUST. More studies are needed to resolve contradictions and obtain clearer results.

Likewise, the WILL-BLEED risk scoring system, developed by Biancari et al. for predicting severe and massive perioperative bleeding in CABG patients, was also found to be reliable in predicting ES transfusion requirements [3]. This scoring system was also found to be more capable of distinguishing potential severe bleeding in a study by Gunertem et al. [12].

While univariate analysis revealed that six of the seven scoring systems (except PAPWORTH) showed significant differences between the ES+ and ES− groups, the predictive performance of these scores was also evaluated using more robust statistical methods. We employed receiver operating characteristic (ROC) curve analysis to assess the discriminatory power of each scoring system in predicting erythrocyte suspension (ES) transfusion. WILL-BLEED, TRUST, and TRACK demonstrated superior sensitivity and specificity in predicting transfusion requirements, which made them stand out as the most reliable models for our cohort. TRUST, for example, is a simpler, more streamlined model compared to other, more complex systems. This simplicity enhances its utility in clinical settings where rapid assessment and decision-making are critical. Similarly, the WILL-BLEED score was specifically designed for coronary artery bypass grafting (CABG) patients and has been validated in multiple studies, which is why it is considered a strong candidate for predicting transfusion requirements. The PAPWORTH score, while showing a high level of significance in our univariate analysis, did not demonstrate sufficient power in our ROC analysis and failed to show significant predictive value for ES transfusion in our cohort. This aligns with previous studies that have questioned its sensitivity in predicting bleeding outcomes in CABG patients, especially when compared to more targeted models like TRUST and WILL-BLEED.

In a study by Klein et al. in 2017 [21], advanced age, presence of DM, low Hb, and high creatinine levels were shown to be associated with a higher risk of blood transfusion. In parallel, Pieri et al. reported that low EF was associated with increased blood transfusion [22]. Another finding of our study was that the ES-receiving patient group had higher age; lower EF, Hb, and Hct; higher creatinine levels; and a greater prevalence of DM.

In a review of 25,000 patients, it was stated that cases with high ES transfusion needs were less frequent, and that the predictive tests primarily focused on identifying these patients. Similarly, in our study, the number of patients requiring 3–4 or more units of ES intraoperatively was limited. However, our strategy aims for a minimal and predictable blood transfusion requirement. Therefore, predicting transfusions of two units or fewer is an important finding. Bianchari et al.’s WILL-BLEED score has been shown to be successful in predicting severe bleeding (>3 units transfused); however, the rate of severe bleeding was 6.4% in that study and 3.25% in our study (13 patients). In both studies, blood transfusions were largely performed in patients with low levels of bleeding. The TRUST score was more successful in identifying these groups [3].

When we examine some scoring systems used to predict bleeding in percutaneous coronary interventions, it becomes evident that the criteria used for perioperative bleeding risk calculation are similarly considered. Baseline hematocrit, creatinine clearance, baseline heart rate, baseline systolic blood pressure, sex, heart failure on admission, prior vascular disease, and diabetes mellitus are some of these variables. These variables are also partially taken into account in the TRACK, TRUST, and WILL-BLEED scores. Therefore, it is not surprising that scores developed for percutaneous interventions also have predictive value in the perioperative setting. In our findings, scores such as ACTION and CRUSADE were found to have predictive power for ES usage. However, they were not superior to the scoring systems specifically designed for cardiac surgery.

Although bleeding risk scores such as CRUSADE and ACTION were originally developed for patients with acute coronary syndromes or undergoing percutaneous coronary intervention (PCI), we included them in our analysis to explore their potential applicability in open-heart surgery. In our study, despite the different contexts in which these scores were developed, both CRUSADE and ACTION scores demonstrated statistically significant predictive value for transfusion requirements, though they were not superior to scoring systems specifically designed for cardiac surgery. Many of the risk factors incorporated in these scores (such as baseline hematocrit, creatinine levels, heart rate, systolic blood pressure, presence of diabetes mellitus, and history of vascular disease) are also relevant for predicting perioperative bleeding and transfusion needs in the cardiac surgery population. However, it is important to note that the hemodynamic, procedural, and pharmacologic characteristics of surgical patients differ significantly from those of acute coronary syndrome (ACS)/PCI populations. For instance, the use of cardiopulmonary bypass, hemodilution, and surgical trauma introduces unique bleeding risks not captured in these scoring systems. Moreover, the perioperative management of anticoagulation and transfusion thresholds is fundamentally different in cardiac surgery. Despite these contextual differences, the inclusion of such scores in our study serves two purposes: (1) to evaluate whether any of their predictive components retain relevance in a surgical setting, and (2) to expose their limitations when applied outside their original population. Our findings support the need for bleeding risk models specifically validated in the cardiac surgery population, which should incorporate surgery-specific variables such as bypass time, surgical technique, and reexploration rates due to bleeding.

In our study, the impact of ES usage on patient outcomes was not a primary focus. This is because the study focused primarily on scoring systems and their ability to predict ES consumption. Another reason is that, since transfusion decisions were already made during surgery, the scoring systems could not have influenced actual patient outcomes in this retrospective analysis. A study investigating whether ES transfusions guided by different scoring systems are associated with adverse outcomes could yield very different results.

This study has several limitations. First, it was conducted retrospectively and is therefore subject to inherent limitations such as selection bias and the potential for missing or incomplete data. Despite our efforts to include all available and consistent data sources (hospital information system, anesthesia records, and patient charts), inaccuracies due to retrospective file review cannot be entirely ruled out. Second, the study was carried out in a single tertiary center, which may limit the generalizability of the results to other institutions with different surgical techniques, patient populations, or perioperative management protocols. Third, although we aimed to minimize confounding by excluding patients with emergent operations, valvular surgery, off-pump procedures, or missing scoring data, other unmeasured confounders (e.g., surgeon experience, intraoperative transfusion thresholds) may still influence the outcomes. In addition, the study period covers 4 years, during which perioperative strategies and transfusion practices may have evolved. Lastly, postoperative bleeding that led to reexploration was not analyzed, which may have limited our ability to comprehensively assess bleeding-related complications.

Current bleeding score calculation methods still have several shortcomings that warrant improvement. These include retrospective data collection, the influence of local and racial variables, and a lack of standardization in surgical procedures or preoperative protocols. However, an ideal evaluation system should be readily accessible, intuitive, and simple to implement.

It is likely that comparing the results of large prospective series for each scoring system would help identify the most effective model in the future. We acknowledge that although we performed ROC curve analyses and logistic regression to evaluate the predictive performance of each risk score, internal validation approaches—such as bootstrapping or k-fold cross-validation—were not employed. This may limit the generalizability of our findings. Future prospective or externally validated studies are required to better assess the robustness of these predictive models. Regardless of the specific system used, it is clear that applying a predictive scoring system can reduce the risks of transfusion, morbidity, and mortality. Measures taken to reduce bleeding and transfusion in cardiac surgery improve the clinical results and reduce costs. Effective blood management is a key component of high-quality care and reduced mortality in cardiac surgery [23].

In conclusion, among the preoperative risk evaluation scoring systems included, WILL-BLEED, TRUST, and TRACK were found to be more predictive of bleeding and the requirement for ES transfusion in CABG operations. Furthermore, low EF, Hb, and Hct levels, higher creatinine levels, and the presence of DM were found to be individual risk factors for perioperative bleeding, independent of the scoring systems. We believe that these scoring systems can be used in research to evaluate intervention outcomes, adjust patient risk, and be incorporated into patient blood management programs.

## Figures and Tables

**Figure:**
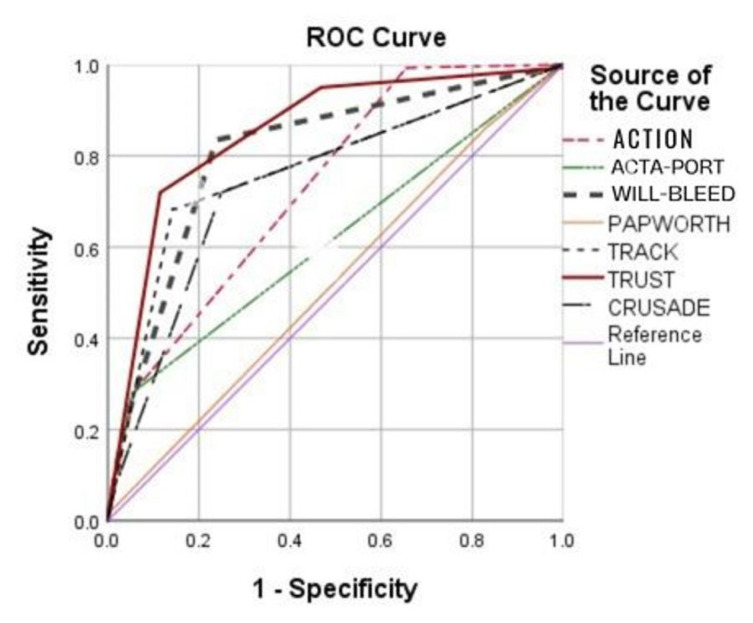
Comparison of risk scores in predicting ES use.

**Table 1:** Demographic data.

	Min–max	Median	Mean±SD
**Age (years)**	33.0–86.0	63	61.81±8.89
**BMI (kg/m** **^2^** **)**	22.0–56.0	30	29.76±4.17
**CC (min)**	22.0–1180	66	65.87±15.01
**CPB (min)**	11.0–165.0	114	113.79±23.63
**EF (%)**	30.0–75.0	55	54.99±7.78
**Hb (g/dL)**	8.0–17.0	13	12.83±1.55
**Hct (%)**	26.0–50.0	39	38.49±4.67
**Creatinine (mg/dL)**	0.57–1.77	0.8	0.85±0.09
	**Frequency (n)**	**Percentage (%)**	
**Sex**			
**Female**	84	21.0%	
**Male**	316	79.0%	
**DM**	215	53.8%	
**HT**	317	79.3%	
**PAD**	5	1.3%	
**CRF**	2	0.5%	
**Anticoagulant use**	13	3.3%	
**ES**			
**0**	261	65.3%	
**I**	73	18.3%	
**II**	53	13.3%	
**III**	12	3.0%	
**IV**	1	0.3%	

BMI: body mass index, CC: cross clamp, CPB: cardiopulmonary bypass duration, EF: ejection fraction, Hb: hemoglobin, Hct: hematocrit, DM: diabetes mellitus, HT: hipertension, PAD: peripheral arterial disease, CRF: chronic renal failure, ES: erythrocyte suspension.

**Table 2:** Comparison of demographic data between patients who received erythrocyte suspension and those who did not.

	ES (−) (n=261)	ES (+) (n=139)	p
**Sex**			0.003*^k^
**Female**	43 (16.5%)	41 (29.5%)	
**Male**	218 (83.5%)	98 (70.5%)	
**Age**	60.13±8.64 (60)	64.96±8.54 (65)	0.001*^m^
**BMI**	29.90±4.19 (30)	29.5±4.14 (30)	0.482^m^
**CC**	65.63±15.63	66.32±13.81	0.665^s^
**CPB**	112.92±24.91	115.41±21.01	0.317^s^
**EF**	56.03±7.58 (55)	53.02±7.79 (55)	0.001*^m^
**Hb**	13.53±1.19 (14)	11.51±1.24 (11)	0.001*^m^
**Hct**	40.60±3.66 (42)	34.53±3.68 (34)	0.001*^m^
**Creatinine**	0.84±0.09 (0.8)	0.86±0.10 (0.9)	0.018*^m^
**DM**	96 (36.8%)	119 (85.6%)	0.001*^k^
**HT**	196 (75.1%)	121 (87.1%)	0.005*^k^
**PAD**	3 (1.1%)	2 (1.4%)	0.804^k^
**CRF**	2 (0.8%)	0 (0%)	0.301^k^
**Anticoagulant**	5 (1.9%)	8 (5.8%)	0.071^k^
**Anemia**	226 (86.6%)	46 (33.1%)	0.001*^k^

s Independent samples t-test: values are given as mean ± standard deviation

m Mann–Whitney U test: values are given as mean ± standard deviation (median)

k Chi-square test: values are given as frequency (percentage)

* p < 0.05: Statistical significance

+BMI: body mass index, CC: cross clamp duration, CPB: cardiopulmonary bypass duration, EF: ejection fraction, Hb: hemoglobin, Hct: hematocrit, DM: diabetes mellitus, HT: hypertension, PAD: peripheral arterial disease, CRF: chronic renal failure, ES: erythrocyte suspension

**Table 3:** Comparison of risk scores between patients who received erythrocyte suspension and those who did not.

		ES (−) (n=261)	ES (+) (n=139)	p
**ACTION**	**0**	90 (34.5%)	1 (0.7%)	0.001*^k^
	**I**	155 (59.4%)	98 (70.5%)	
	**II**	16 (6.1%)	38 (27.3%)	
	**III**	0 (0%)	2 (1.4%)	
**ACTA-PORT**	**0**	1 (0.4%)	0 (0%)	0.001*^k^
	**I**	246 (94.3%)	100 (71.9%)	
	**II**	14 (5.4%)	36 (25.9%)	
	**III**	0 (0%)	3 (2.2%)	
**WILL-BLEED**	**I**	199 (76.2%)	23 (16.5%)	0.001*^k^
	**II**	53 (20.3%)	88 (63.3%)	
	**III**	9 (3.4%)	27 (19.4%)	
	**IV**	0 (0.0%)	1 (0.7%)	
**PAPWORTH**	**I**	44 (16.9%)	19 (13.7%)	0.112^k^
	**II**	217 (83.1%)	118 (84.9%)	
	**III**	0 (0.0%)	2 (1.4%)	
**TRACK**	**I**	222 (85.1%)	44 (31.7%)	0.001*^k^
	**II**	2 (0.8%)	0 (0.0%)	
	**III**	37 (14.2%)	95 (68.3%)	
**TRUST**	**0**	1 (0.4%)	1 (0.7%)	0.001*^k^
	**I**	138 (52.9%)	6 (4.3%)	
	**II**	92 (35.2%)	32 (23.0%)	
	**III**	30 (11.5%)	99 (71.2%)	
	**IV**	0 (0.0%)	1 (0.7%)	
**CRUSADE**	**0**	196 (75.1%)	39 (28.1%)	0.001*^k^
	**I**	59 (22.6%)	87 (62.6%)	
	**II**	6 (2.3%)	12 (8.6%)	
	**III**	0 (0.0%)	1 (0.7%)	

k Chi-square test: values are given as frequency (percentage).

* p < 0.05: the level of statistical significance.

**Table 4:** Comparison of predictive values of risk scores in predicting ES use.

	Area under curve	95% confidence interval	p
ACTION	0.733	0.685–0.782	0.001
ACTA-PORT	0.615	0.555–0.676	0.001
WILL-BLEED	0.808	0.762–0.854	0.001
PAPWORTH	0.522	0.463–0.581	0.470
TRACK	0.770	0.718–0.822	0.001
TRUST	0.853	0.814–0.893	0.001
CRUSADE	0.739	0.686–0.791	0.001

**Table 5:** Independent predictors of erythrocyte suspension use.

	Single variable model			Multiple variable model		
	OR	95% confidence interval	p	OR	95% confidence interval	p
**Age**	1.07	1.04–1.09	0.001			
**EF**	0.95	0.93–0.98	0.001			
**Sex**	0.47	0.29–0.77	0.003			
**Hb**	0.29	0.24–0.37	0.001			
**Hct**	0.67	0.62–0.73	0.001			
**DM**	10.23	5.98–17.49	0.001	5.20	2.28–11.89	0.001
**HT**	2.23	1.26–3.94	0.006			
**Anticoagulant**	3.13	1.01–9.75	0.049			
**ACTION**	7.49	4.47–12.55	0.001	2.79	1.02–7.64	0.045
**ACTA-PORT**	6.60	3.47–12.54	0.001			
**WILL-BLEED**	8.09	5.24–12.47	0.001	2.00	1.06–3.78	0.033
**TRACK**	3.59	2.80–4.61	0.001			
**TRUST**	8.16	5.48–12.13	0.001	2.56	1.34–4.88	0.004
**CRUSADE**	5.45	3.60–8.25	0.001			

Logistic regression analysis

**Appendix:** The scoring systems used in the study.

NAME OF THE SCORING SYSTEM	DEFINITION	PARAMETERS INCLUDED	RISK CLASSIFICATION
**ACTION (7,11)**	Acute Coronary Treatment and Intervention Outcomes Network	Age, basal serum creatinine, systolic blood pressure on admission, basal hemoglobin, pulse rate on admission, sex, body weight, warfarin usage, diabetes mellitus, heart failure or cardiogenic shock on admission, electrocardiographic changes, presence of peripheral arterial disease	0: ≤20 (very low risk), I: 21–30 (low risk), II: 31–40 (moderate risk), III: 41 to 50 (high risk), IV: ≥50 (very high risk)
**ACTA-PORT(12)**	The Association of Cardiothoracic Anesthetists (ACTA) perioperative risk of blood transfusion score	Age, sex, body surface area, logistic EuroSCORE, preoperative hemoglobin, and creatinine, type of operation	0: 0–14 (low risk), I: 15–19 (moderate risk), II: 20–24 (high risk), III: 25–30 (very high risk)
**WILL-BLEED(3,5,14)**	Used to predict severe and massive perioperative bleeding in patients undergoing CABG.	Usage of low molecular weight heparin/fondaparinux/unfractioned heparin, duration of pause of a potent antithrombotic drug, sex, acute coronary syndrome, anemia, eGFR, presence of critical preoperative state	I: 0–3 (low risk), II: 4–6 (moderate risk), III: >6 (high risk)
**PAPWORTH(12)**	High, moderate, and low risk of postoperative bleeding	Surgical priority, type of surgery, valvular aortic disease, body mass index, age	I: 0 (low risk), II: 1–2 (moderate risk), III: ≥3 (high risk)
**TRACK(13)**	Transfusion risk and clinical knowledge	Age, body weight, sex, complex of surgery, hematocrit level	I: <13 (low risk), II: >13 (high risk)
**TRUST (13, 14)**	Transfusion Risk Understanding Scoring Tool	Age, body weight, sex, preoperative hematocrit, preoperative Hb, preoperative creatinine, presence of previous cardiac surgery, complex surgery (Coronary revascularization + valve surgery, valve surgery, reoperations and surgery of the ascending aorta were named as complex surgery)	0: 0–1 (low risk), I: 2 (moderate risk), II: 3 (high risk), III: 4–8 (very high risk)
**CRUSADE(6, 15)**	Can Rapid Risk Stratification of Unstable Angina Patients Suppress Adverse Outcomes With Early Implementation of the ACC/AHA Guidelines	Basal hematocrit, creatinine clearance, basal heart rate, basal systolic blood pressure, sex, heart failure on admission, prior vascular disease, diabetes mellitus	0: ≤20 (very low risk), I: 21–30 (low risk), II: 31–40 (moderate risk), III: 41 to 50 (high risk), IV: ≥50 (very high risk)

* Group IV does not exist in ACTION and CRUSADE since these do not have “very high risk” patient class.

## Abbreviations

ES: erythrocyte suspension
TRACK: Transfusion Risk and Clinical Knowledge
PAPWORTH: The Papworth Bleeding Risk Score
WILL-BLEED: Bleeding prediction score CRUSADE
ACTION: Acute Coronary Treatment and Intervention Outcomes Network
TRUST: Transfusion Risk Understanding Scoring Tool
ACTA-PORT: The Association of Cardiothoracic Anesthetists (ACTA) perioperative risk of blood transfusion score
